# A causal model of human growth and its estimation using temporally sparse data

**DOI:** 10.1098/rsos.250084

**Published:** 2025-08-07

**Authors:** John A. Bunce, Catalina I. Fernández, Caissa Revilla-Minaya

**Affiliations:** ^1^ Division of Anthropology, American Museum of Natural History, New York, NY, USA; ^2^ Department of Human Behavior, Ecology, and Culture, Max Planck Institute for Evolutionary Anthropology, Leipzig, Sachsen, Germany; ^3^ Department of Anthropology, Florida Atlantic University, Boca Raton, FL, USA

**Keywords:** Growth, Ontogeny, Metabolism, Allometry, Amazonia, Indigenous Peoples

## Abstract

Existing models of human growth provide limited insight into underlying mechanisms responsible for inter-individual and inter-population variation in children’s growth trajectories. Building on general theories linking growth to metabolic rates, we develop a causal parametric model of height and weight growth incorporating a representation of human body allometry and a process-partitioned representation of ontogeny. This model permits separation of metabolic causes of growth variation, potentially influenced by nutrition and disease, from allometric factors, potentially under stronger genetic control. We estimate model parameters using a Bayesian multilevel statistical design applied to temporally dense height and weight measurements of U.S. children, and temporally sparse measurements of Indigenous Amazonian children. This facilitates a comparison of the contributions of metabolism and allometry to observed cross-cultural variation in the growth trajectories of the two populations, and permits simulation of the effects of healthcare interventions on growth. This theoretical model provides a new framework for exploring the causes of growth variation in our species, while potentially guiding the development of appropriate, and desired, healthcare interventions in societies confronting growth-related health challenges, such as malnutrition and stunting.

## 1. Introduction

Between birth and adulthood, humans generally increase in height and weight following trajectories unique to our species, and notably different from those of most other mammals [1–3]. However, like many widely distributed animals, the typical adult body form, and thus the typical shape of the growth trajectory, differs between populations in different parts of the world [4]. Some aspects of body form are heavily influenced by genes [5,6]. Thus, both drift and natural selection may contribute to this intra-species variation through their action on genes at the population level over generations in different terrestrial ecosystems (e.g. Allen’s and Bergmann’s Rules: [7]). Other important contributions to variation in human body form come from environmental factors, such as food availability, pathogen exposure and chronic stress [8–11], that directly, and differentially, affect the growth of individuals in different societies around the world. Distinguishing among these contributions to population-level variation in growth is a major challenge [12], both for our understanding of human ontogeny, and for the provision of appropriate healthcare support to individuals whose particular patterns of growth result from exposure to health challenges that can be alleviated (e.g. stunting and wasting as indicators of malnutrition: [13,14]). Here, we present a new approach to exploring these different causes of variation by deriving a model of human growth from fundamental principles of metabolism and allometry.

### 1.1. Growth models

Over the last century, auxology, the study of human growth, has produced a rich set of mathematical models to describe the complex growth trajectories of children. Growth models are often classified as ‘parametric’ or ‘non-parametric’. Parametric models define a specific range of possible forms for the growth trajectory, implemented as a mathematical relationship between a (relatively small) number of parameters, the values of which (and the shape of the resulting trajectory) may vary from person to person. Ideally, the mathematical relationship is derived from some causal theory of growth, and the parameter values thus have a mechanistic interpretation. However, for most popular parametric models (e.g. [15]), generally neither is true [16,17]. Non-parametric models, e.g. splines, are explicitly descriptive, and can be made to fit growth data arbitrarily well. However, their (generally numerous) parameters are, by design, biologically uninterpretable, offering no causal insight. A third class, called ‘shape invariant’ models, employ either a parametric (e.g. QEPS: [18]) or non-parametric (e.g. SITAR: [19]) function to represent the population mean trajectory, and then use a smaller set of individual-level parameters to account for each individual’s deviation from this mean trajectory [17]. All of these models are valuable tools for representing and comparing individual growth trajectories given the temporal resolution of most available data (where higher resolution data motivates structurally different models: [20,21]). Importantly, however, none of these models is derived from a causal theory of human growth, i.e. a theory that identifies biological quantities measurable independently of growth, and proposes a relationship between them that causes an observable growth trajectory. Existing models, therefore, provide limited insight into the mechanisms through which individual characteristics (e.g. genes) and experiences (e.g. diet, illness) interact to produce an observed pattern of growth.

Though rarely applied specifically to humans, general causal models of organismal growth have a long history in biology. Pütter [22] proposed a theory, further developed and popularized by von Bertalanffy [1,23], such that organisms grow when the energy released by breaking down molecules acquired from the environment (via catabolism) exceeds the energy required for homeostasis (e.g. cellular maintenance). The excess energy can be used to synthesize new structural molecules (via anabolism) using both the products of catabolism and other substrates acquired from the environment. Because all living cells perform catabolic reactions, an organism’s total catabolic rate is a function of its mass. However, in an environment with abundant energy-containing substrates, the production of the excess energy required for anabolic structural growth is a function of the surface area of the body’s interface with the environment through which structural and energy-containing molecules are absorbed. In recent years, this basic principle has been incorporated into more general theories, detailing how environmental substrates of a given energy density, acquired by cells and transformed into reserves, are then allocated to maintenance, growth and reproduction [24,25], how anabolic and catabolic processes (and therefore growth) depend on temperature [26], and how anabolism (and therefore growth) is limited by the structure of the supply network (e.g. capillaries) transporting environmental substrates to internal cells [27,28] (see also electronic supplementary material, appendix D.5.1). We develop a causal theory of growth tailored to humans by combining the basal model of Pütter [22] and von Bertalanffy [23] with a representation of human body allometry, and a process-partitioned representation of human ontogeny. This results in a new parametric model of the metabolic processes and allometric relationships that underlie both the unique species-typical pattern, and the individual variations, of human growth.

### 1.2. Model estimation

We apply our new model to height and weight measurements from two populations that, on average, exhibit different patterns of growth: affluent U.S. children born in the late 1920s and Indigenous Matsigenka children from Amazonian Peru measured in 2017−2019. Importantly, these two datasets also differ in temporal resolution: each U.S. child was measured (at least) annually from birth to age 18 [29], while most Matsigenka were measured (by authors C.R.M. and J.A.B.) only once or twice between the ages of one and 24. We believe the temporal sparsity of the Matsigenka dataset is typical of data feasibly collected in many rural, isolated or marginalized societies around the world. Attention to such societies is essential, both to understand the range of growth variation in our species, and, even more importantly, to provide desired and appropriate support to address growth-affecting health challenges faced by many of these populations.

To accommodate the differing temporal resolutions of the two datasets, we design a Bayesian multilevel (mixed-effects) estimation strategy [30–32]. Given our theoretical model, this allows us to quantify differences between U.S. and Matsigenka children in metabolic rates, plausibly influenced by population-level differences in diet and/or disease exposure, as well as differences in allometric relationships, potentially (though not necessarily: [9]) under stronger genetic influence. Additionally, by modifying a fit model’s metabolic parameters, we can simulate population-specific effects on growth of a healthcare intervention affecting metabolic rates, such as dietary supplementation and/or disease reduction. An important goal of this approach is a tool to suggest when, and what form of, healthcare interventions would increase child wellbeing in a particular population, using data feasibly collected across a broad range of human societies.

## 2. Methods

### 2.1. Derivation of the theoretical growth model

In the Pütter [22] and von Bertalanffy [23] theory of growth, mass increases when the rate of anabolism (using energy to construct molecules) is greater than the rate of catabolism (breaking down molecules to release energy). All cells perform catabolic reactions. Anabolism is contingent on the acquisition of molecules (containing both energy and building materials) from the environment. Only parts of the organism in contact with the environment can acquire such molecules. Therefore, anabolism is a function of an organism’s absorbing surface area (under the assumption of abundant food: [24]), while catabolism is a function of an organism’s mass:


(2.1) $$\frac{dm}{dt}=Hs-Km$$

where m is mass, s is surface area, H≥0 is synthesized mass (i.e. substrate molecules chemically bonded via anabolism) per unit of an organism’s absorbing surface, and K≥0 is destructed mass (i.e. molecules whose chemical bonds are broken via catabolism) per unit of an organism’s mass.

We are interested in the rate of longitudinal (i.e. height) growth per unit time. The shape of the body is stylized as a cylinder with height h, radius r and substrate-absorbing (i.e. intestinal) surface area 2πrh (electronic supplementary material, appendix A, with modification in appendix D.1.2). Equation (2.1) can be used to approximate the rate of growth in mass ( = volume ⋅ density):


(2.2) $$\begin{array}{lrlr} & \frac{d}{dt}D\pi r^{2}h & =H(2\pi rh)-K(D\pi r^{2}h) & \end{array}$$

where D is density. Solving equation (2.2) for the longitudinal growth rate dh/dt yields:


(2.3) $$\frac{dh}{dt}=\frac{2H}{D}\frac{h}{r}-Kh-\frac{2h}{r}\frac{dr}{dt}.$$

We assume a power law relationship between r and h such that $r=h^{q}$, where 0<q<1 represents the allometric relationship between radius and height as the body grows in both dimensions (electronic supplementary material, appendix A). Substituting for r in equation (2.3) yields:


(2.4) $$\frac{dh}{dt}=h^{1-q}\left(\frac{2H}{D(1+2q)}-\frac{K}{1+2q}h^{q}\right).$$

Under the same assumptions (electronic supplementary material, appendix B), re-writing s in equation (2.1) in terms of m yields the rate of growth in mass:


(2.5) $$\begin{array}{lrlr} & \frac{dm}{dt} & =\frac{2H}{D}(D\pi {)}^{\frac{q}{2q+1}}\cdot m^{\frac{q+1}{2q+1}}-Km & \end{array}$$

Assuming D=1 g/cm${,}^{3}$, solving differential equations (2.4) and (2.5) for h(t) and m(t) yields:


(2.6)h(t)=[2HK(1−eKq1+2q(i−t))]1q(2.7)m(t)=π[2HK(1−eKq1+2q(i−t))]1q+2

for all t≥i, where t is total age in years since conception, and i≥0 is the age at which growth is initiated, such that, for growth in early life, i=0 at conception. Detailed derivations and solutions, as well as justifications for assumptions, are provided in electronic supplementary material, appendices A and B.

It is well-established that different tissues in the human body, e.g. bones of the leg versus torso, grow with different acceleration profiles, attaining high growth velocities at different ages during ontogeny ([4], pg 35, 82). There is also evidence that groups of tissues may undergo coordinated cycles of faster and slower growth on time scales of several years [33], several months [34] and several days [35,36], likely due to hormonal regimes whose effects and interactions are still poorly understood (electronic supplementary material, appendix C). The additive effect of these partially overlapping growth processes contributes to the complexity of the human growth trajectory, such that greater complexity (e.g. frequency of changes in the sign of acceleration) becomes more apparent the higher the temporal resolution of measurements.

In principle, equations (2.6) and (2.7) are general enough to apply at any time scale to any growing tissue linked via blood vessels to the intestinal surface. Total height (or weight) can thus be modeled as the sum of multiple equations (2.6) or (2.7), where each component equation represents one cycle of the growth of a particular tissue (or group of tissues). The number of components to add together within such a composite model is as much an empirical as a theoretical question, and depends on the temporal resolution of the data to which the model will be fit, as well as the expected measurement error. Here, we construct a composite model with five additive components (justified in electronic supplementary material, appendix C). Each component function may have different values for the parameters H, K, q, and i, represented by subscripts 1 to 5. This yields the following 20-parameter composite growth models for height (equation (2.8)) and weight (equation (2.9)) at total age t:


(2.8)h(t)=∑x=15[t≥ix][2HxKx(1−eKxqx1+2qx(ix−t))]1qx(2.9)m(t)=∑x=15[t≥ix]π[2HxKx(1−eKxqx1+2qx(ix−t))]1qx+2

such that $i_{1}=0$ represents conception, and $\left[t\geq i_{x}\right]$ is Iverson notation [37], taking the value of one if the condition within brackets evaluates to True, and is zero otherwise.

**Figure 1.**
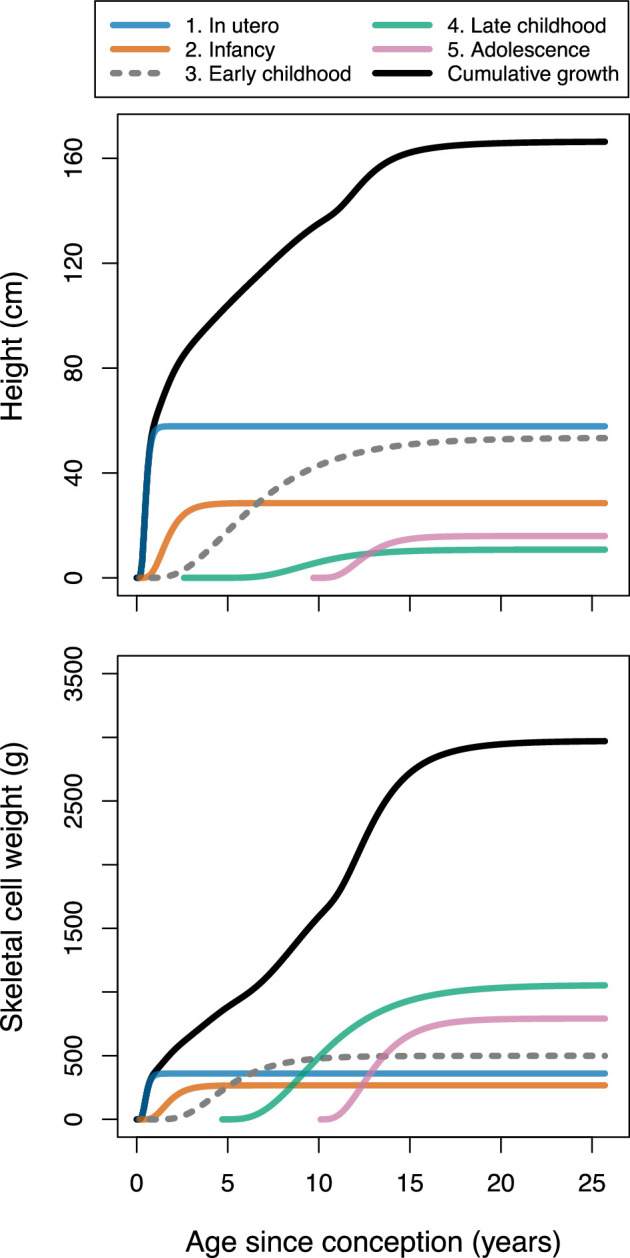
Illustration of the composite growth model comprising the sum of five component growth processes, fit to height and skeletal cell weight (electronic supplementary material, appendix D.1.1) of U.S. girls. Height (upper) and weight (lower) plots are generated from two variants of the model, explained in electronic supplementary material, appendices D.3 and F.1, respectively.

Figure 1 presents a graphical illustration of the composite growth model for height and weight. For convenience, the five component processes are labelled according to the ontogenetic phase in which each has the largest effect on overall growth. The relative positions of the five component functions with respect to age are estimated empirically (with guidance from priors) through fitting the model, and are not fixed *a priori*. Thus, use of this growth model does not require us to define the five growth processes in terms of age. For illustration, the height and weight plots in figure 1 are generated with different model fitting parameters to better match each type of growth data (described in electronic supplementary material, appendices D.3 and F.1, respectively). As explained in electronic supplementary material, appendix D.1.1, the model is fit to skeletal cell weight rather than total body weight. Electronic supplementary material, appendix figures A.5 and A.6 show how modifying values of parameters q, K, H and i changes the shape of a component growth trajectory.

### 2.2. Empirical analysis

We fit the composite growth model equations (2.8) and (2.9) to two datasets: (i) temporally dense height and weight measures from 70 girls and 66 boys born in 1928 and 1929 to parents of relatively high socioeconomic status in Berkeley, California, USA [29]; and (ii) temporally sparse measures from 196 girls and 179 boys collected by C.R.M. and J.A.B. between 2017 and 2019 in four Matsigenka Native Communities inside Manu National Park in the lowland Amazonian region of Peru. Matsigenka in these communities are horticulturalists, gatherers, fishers and hunters who produce most of their own food and have relatively little (though increasing) dependence on broader Peruvian society [38–40]. We refer to these as the U.S. and Matsigenka datasets, respectively.

The U.S. dataset comprises data collected at regular intervals between birth and (at most) 21 years of age (shorter intervals before age two), with an average of 30 measurements per individual. The original authors then manually smoothed the data by taking a moving weighted average of the actual measurements ([29], pg 193−198). Additional extrapolated measurements reported in the original dataset are excluded. To aid in fitting the theoretical model, we duplicated each individual’s last reported height and weight measurements yearly until the age of 26. We also set each individual’s height to 0.012 cm and weight to $1.02\cdot 10^{-6}$ g (i.e. that of an egg cell: [41,42]) at nine months prior to birth (i.e. conception).

The Matsigenka dataset comprises data collected using an electronic balance (Tanita BC−351) and a stadiometer (Seca 213) from all individuals who were between 2 and 24 years of age and residing in the Matsigenka communities or attending one of three boarding secondary schools in neighboring communities at the time of our visits between 2017 and 2019. One Matsigenka community and the three boarding schools were visited twice in this period. Five one-year-old children whose parents were eager for them to be measured are also included in the dataset. Due to our uncertainty about age in this population, ages are recorded to the nearest year. While uncertainty in age is not modeled in the analyses below, a preliminary exploration of the effect of variance in recorded age on model estimates is presented in electronic supplementary material, appendix D.6. A maximum of 3, and an average of 1.3, measurements per person were collected. The majority (70%) of Matsigenka children are represented by measurements at a single time point. As with the U.S. dataset, we set each individual’s height and weight to that of an egg cell at conception. Raw data are plotted in electronic supplementary material, appendix figure A.8, with dataset characteristics in electronic supplementary material, appendix table A.2. Application of the model to cross-sectional datasets is discussed in electronic supplementary material, appendix D.7.

To obtain more accurate parameter estimates (electronic supplementary material, appendix D.1.3), we simultaneously fit the model to both height and weight data in the combined U.S. and Matsigenka datasets. Observed height $h_{jt}$ and weight $m_{jt}$ of person j at total age t years since conception are given by:


(2.10)ln⁡(hjt)∼Normal[ln⁡(0.012+ηjt),ση](2.11)ln⁡(mjt)∼Normal[ln⁡(1.02⋅10−6+μjt),σμ]

where $\eta_{jt}$ and $\mu_{jt}$ are equations (2.8) and (2.9), respectively, which, when each parameter is indexed by j, represent height and weight trajectories of individual j in the five growth processes. These equations are added to the height (diameter) and weight of a human ovum. To represent the fact that observed heights and weights can never be negative, we take the log of both the observations and the mean of the Normal likelihood. The standard deviations $\sigma_{\eta }$ and $\sigma_{\mu }$ represent measurement error, on the log scale, around individual j’s actual height and weight, respectively, at total age t. Females and males are fit with separate models.

We use a multilevel model design in a Bayesian framework [30], such that individuals’ height or weight trajectories in a given component growth function are estimated as the sum of ethnic group-level and person-level offsets to a baseline growth trajectory (i.e. random effects). We allow these offsets to covary across growth processes of an individual. Complete model structure, derivation and priors are provided in electronic supplementary material, appendix D. Models were fit in R [43] and Stan [44] using the cmdstanr package [45]. Data and analysis scripts are provided at https://github.com/jabunce/bunce-fernandez-revilla-2022-growth-model. The robustness of results to changes in the informative priors is explored in electronic supplementary material, appendix D.8. After fitting the model, posterior parameter estimates are systematically modified to simulate the effects on growth of specific healthcare interventions likely to affect metabolism.

## 3. Results

### 3.1. Theoretical model comparison

The composite model presented here was compared against the parametric JPA-1 model of Jolicoeur *et al*. [15] (electronic supplementary material, appendix F.3) and the spline-based shape invariant SITAR model of Cole *et al*. [19] (electronic supplementary material, appendix F.4) by fitting all three models to measurements of U.S. boys. As described above, the composite model was fit simultaneously to heights and weights (electronic supplementary material, appendix D.1.3), while the latter two models were fit only to height, as they are not designed to be fit to both data types simultaneously. The fact that these models are fit to different datasets precludes their comparison using metrics of out-of-sample predictive accuracy, such as WAIC (widely applicable information criterion), LOOCV (leave-one-out cross validation) and PSIS (Pareto-smoothed importance sampling), that are computed point-wise [(30), pg 220]. Qualitative visual inspection suggests that estimates of the mean height trajectory by the JPA-1 and SITAR models are more similar to each other than they are to estimates of the composite model (electronic supplementary material, appendix figure A.28). However, estimates of all three models generally coincide well, giving us confidence that the composite model is appropriate for population-level comparisons of growth. Detailed qualitative model comparison and analysis of residuals are presented in electronic supplementary material, appendix G.

### 3.2. Empirical estimates

**Figure 2.**
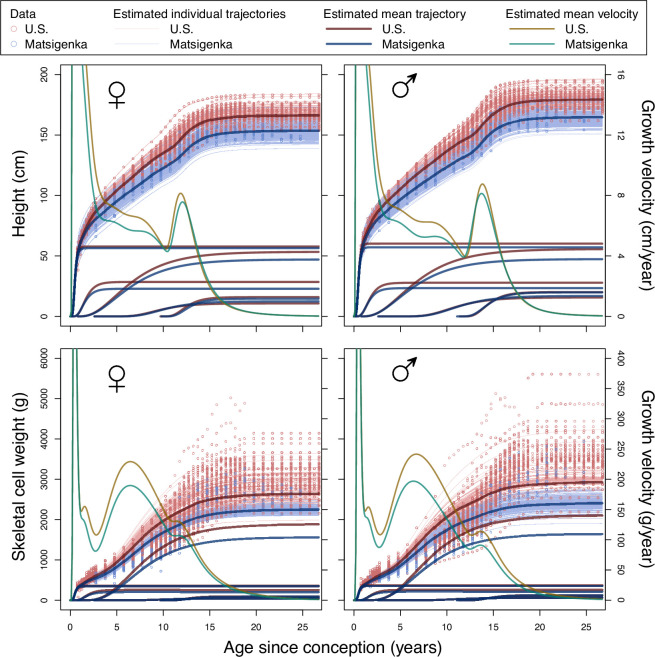
Posterior estimates from the composite growth model fit to U.S. (red) and Matsigenka (blue) height and weight. Thin lines are mean posterior trajectories for each individual. Thick red and blue lines are mean posteriors for the mean cumulative trajectories of each ethnic group, as well as the mean trajectories for each of the five component growth processes. Corresponding mean posterior mean cumulative velocity trajectories in green and orange are decreasing (after age 14) from left to right.

Figure 2 shows differences in mean posterior estimates of population-level and individual-level height and weight trajectories for U.S. and Matsigenka girls and boys between conception and adulthood. Note that estimated height trajectories fit the measurement data better than estimated weight trajectories. This is by design, as explained in electronic supplementary material, appendix D.1.3. Comparing trajectories of the five component processes across U.S. and Matsigenka girls and boys, the model predicts that population-mean differences in overall height and weight are primarily the result of processes beginning during infancy and early childhood (and, for boys, *in utero*). Electronic supplementary material, appendix figure A.10 incorporates uncertainty in the estimated U.S. and Matsigenka mean growth trajectories in order to compute descriptive characteristics of these trajectories: maximum achieved height, maximum achieved growth velocity during puberty, and the age at maximum velocity during puberty. Analysis of residuals is presented in electronic supplementary material, appendix G.2.

**Figure 3.**
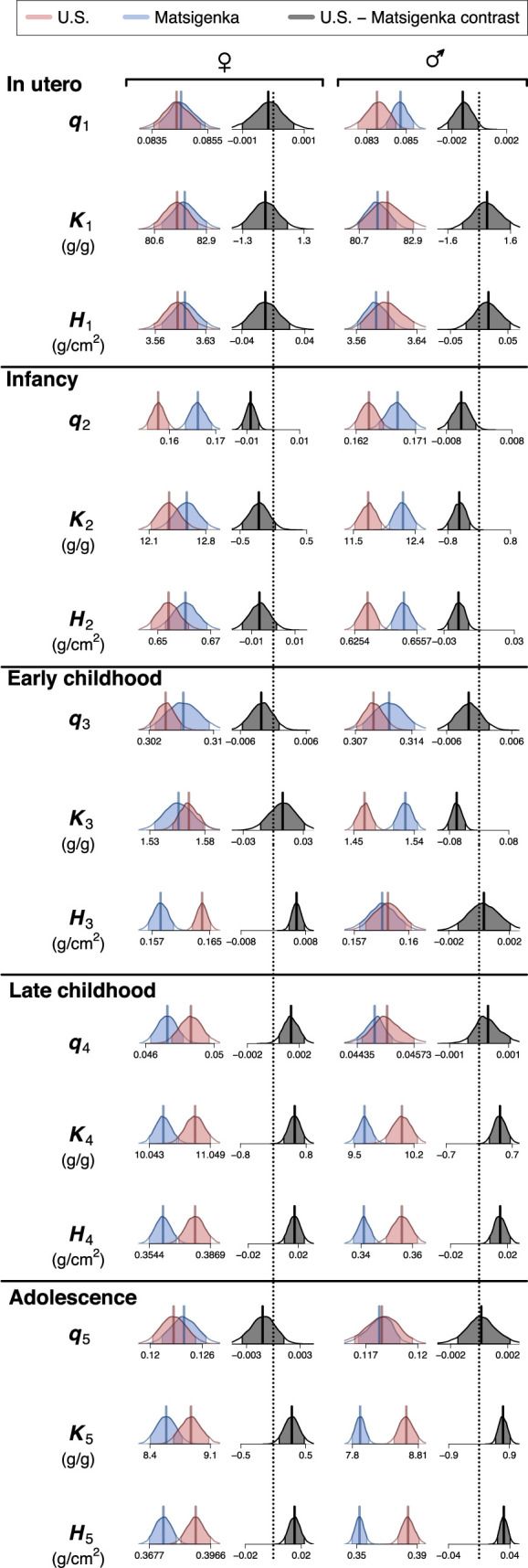
Estimates of mean parameter values by ethnic group. Shown are 90% highest posterior density intervals (HPDIs: [30]) for posterior distributions of model parameters used to construct the mean height and weight trajectories (figure 2) of U.S. (red) and Matsigenka (blue) children, by growth process. Posterior density outside of this HPDI is shown as white tails on a distribution. Distribution means are shown as solid vertical lines. The 90% HPDI of the U.S.—Matsigenka contrast (difference) is shown in grey. HPDIs of contrasts that do not overlap zero (dotted vertical lines) indicate a detectable ethnic-group difference in parameter estimates.

**Figure 4.**
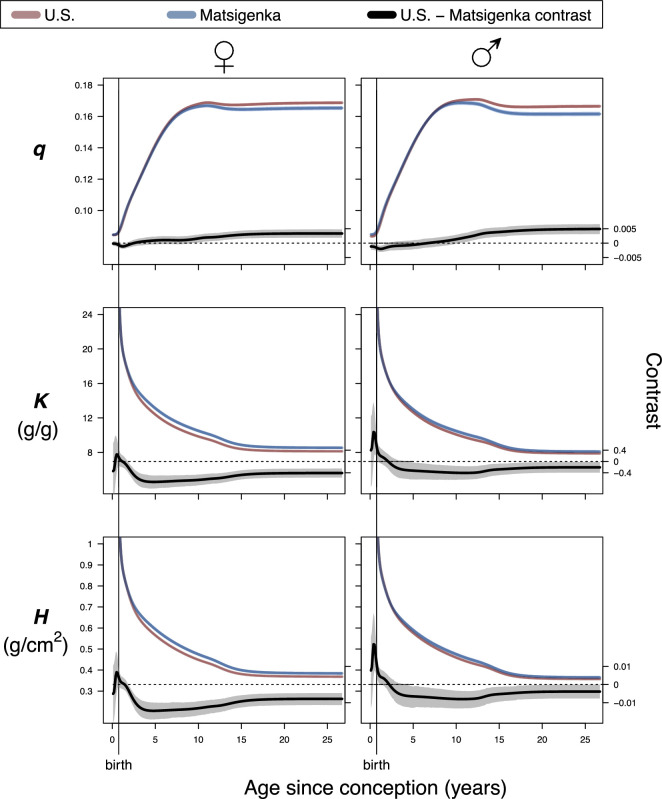
Estimates of mean parameter values by age for each ethnic group. Lines are means and shaded areas are 90% highest posterior density intervals (HPDI) for posterior distributions of sums of model parameters q (allometry), K (catabolism) and H (anabolism) across the five growth processes (figure 1) at a given age, weighted by the contribution of each process to overall height and metabolism at that age (electronic supplementary material, appendix D.4), for U.S. (red) and Matsigenka (blue) children. HPDIs for parameter trajectories are often too narrow to be visible around the mean lines. The 90% HPDI of the U.S.—Matsigenka contrast (difference) is shown in grey. HPDIs of contrasts that do not overlap zero (dotted horizontal lines) indicate a detectable ethnic-group difference in parameter estimates.

Figure 3 presents a comparison of population-mean parameter estimates for U.S. and Matsigenka children, for each of the five growth processes in figure 1 (i parameters are shown in electronic supplementary material, appendix figure A.9). Note that uncertainty in estimates of Matsigenka population mean parameters is comparable to uncertainty in U.S. population mean parameters, despite the temporal sparsity of measurements for each Matsigenka individual (see also electronic supplementary material, appendix G.2). Parameter estimates are often readily distinguishable between the two ethnic groups, though patterns often differ by sex. However, because the five growth processes overlap in time, interpreting metabolism and allometry of the body as a whole requires a strategy for combining (summing) these process-specific parameter estimates. In figure 4, population-mean parameter estimates are summed for each age over the entire period of growth. For a given age, parameters in each of the five growth processes are weighted by the contribution of that process to overall height. Metabolic parameters K and H are further weighted by an expected decrease in metabolic activity as each process approaches its asymptote (electronic supplementary material, appendix D.4). This analysis suggests that, during most of ontogeny, Matsigenka girls and boys tend to have lower values of the allometric parameter q (i.e. smaller intestinal surface area, and concomitant body circumference, for a given height), and higher rates of catabolism (K) and anabolism (H) than U.S. children. These trends mark a reversal from the perinatal period. Objective values of metabolic parameters K and H shown in figure 4 coincide reasonably well with estimates of catabolic and anabolic rates calculated independently from published U.S. government data on average contemporary dietary intake in the national population (electronic supplementary material, appendix D.5). Such dietary intake data have yet to be collected for the Matsigenka population.

### 3.3. Intervention simulation

**Figure 5.**
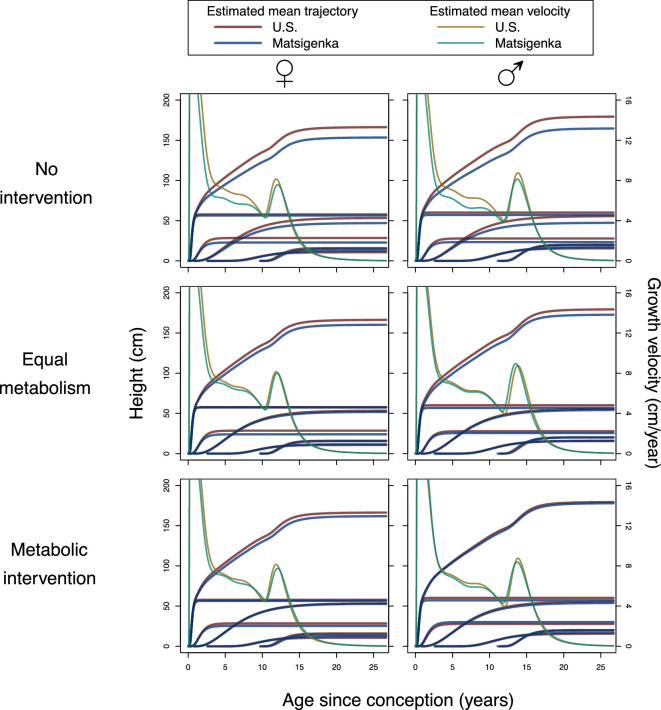
Simulated effects of healthcare interventions on Matsigenka mean height. Shown are mean posterior trajectories for mean overall height and five component growth processes for U.S. (red) and Matsigenka (blue) children. Corresponding mean growth velocities are decreasing (after age 14) from left to right. The first row reproduces figure 2, and represents the pre-intervention state. In the second row, values of all metabolic parameters (K's and H's) for Matsigenka are modified to match mean estimates of those for U.S. children. In the third row, only Matsigenka values of $K_{2}$ and $H_{3}$ (girls) and $K_{2}$ and $K_{3}$ (boys) are modified to match mean estimates of those for U.S. children. Further analysis of interventions is presented in electronic supplementary material, appendix E.

Figure 5 shows the predicted effects of two metabolic interventions on mean Matsigenka height trajectories. In the first intervention (second row of figure 5), values of all Matsigenka metabolic parameters (H’s and K’s) are changed to match the mean estimates for U.S. children. This allows us to see the contribution of allometry (q parameters) to the observed mean height differences between Matsigenka and U.S. children.

The second is a targeted metabolic intervention (third row of figure 5) meant to simulate dietary supplementation (presumed to increase H) and a decrease in immune system activation due to disease (presumed to decrease K) in the Matsigenka population during infancy and early childhood (the second and third component growth processes: figure 1). As shown in figure 3, in the infancy process, both Matsigenka girls and boys are estimated to have higher mean values of $K_{2}$ and $H_{2}$ than U.S. children. Thus, we decreased $K_{2}$ for Matsigenka girls and boys to match that of U.S. girls and boys, but did not modify $H_{2}$. In the early childhood process, Matsigenka girls are estimated to have lower mean $H_{3}$ and indistinguishable $K_{3}$ compared to U.S. girls, while Matsigenka boys were estimated to have higher mean $K_{3}$ and indistinguishable $H_{3}$ compared to U.S. boys. Thus, we increased $H_{3}$ for Matsigenka girls to match that of U.S. girls, and decreased $K_{3}$ for Matsigenka boys to match that of U.S. boys. Because diet and disease are probably not solely responsible for population-level differences in average metabolism (e.g. genes may also contribute), these simulated changes likely represent an upper limit on the effect of an intervention targeting diet and disease.

Comparing maximum achieved height in the first row of figure 5 against that in the last two rows suggests that, under both interventions, Matsigenka children would grow taller. However, with the possible exception of boys in the second intervention, Matsigenka children are still predicted to be shorter than U.S. children, on average. This suggests that allometric (i.e. body shape) differences, represented by the q parameters, are at least partly responsible for observable mean height differences between these two populations. Electronic supplementary material, appendix E presents additional analyses of these interventions, including their effects on weight, and shows how modifying metabolic parameters H and K can contribute to changing overall body allometry, as well as the age at maximum growth velocity, even if q and i parameters are unmodified. Note that these intervention simulations are idealized, in that the value of each H and K metabolic parameter is assumed to be modifiable independently of the others, and independently of the allometric q parameters. Electronic supplementary material, appendix figure A.6 demonstrates that covariance among H and K, such that the quantity H/K is constant, can have unique effects on the growth trajectory. Similarly, §4.3: Healthcare interventions, below, describes how changes in metabolic parameters H and K can affect body-wide calculations of the allometric parameter q. Thus, predictions of simulated healthcare interventions, where H and K are independently varied, may diverge from consequences of real-world interventions, where such variables may covary in ways that are not yet known.

## 4. Discussion

We have developed a new causal model of human growth and estimated its parameters using temporally dense data from a population of U.S. children, and temporally sparse data from a population of Indigenous Matsigenka children. The model predicts that these two ethnic groups differ in terms of overall mean rates of anabolism and catabolism, and the allometric relationship between body height and intestinal surface area. Furthermore, analysis of the model suggests that both metabolism and allometry contribute to observed mean size differences between the populations. Below we present possible explanations for these differences, implications for health interventions, limitations of this new approach and future directions.

### 4.1. General trends

Figure 4 suggests that total rates of catabolism (K) and anabolism (H) are very high at conception and then decrease towards an asymptote by age 20 (resembling a horizontal reflection of height growth: figure 2). This general pattern can be compared to the findings of Pontzer *et al*. [46], who use a doubly labeled water method with international participants of a range of ages to calculate size-adjusted total daily energy expenditure (TEE), conceptually similar to catabolic rate K. Adjusted TEE at birth resembles that of adults, but increases rapidly during the first year, after which it declines toward an asymptote at age 20. At birth, the relatively low estimate of TEE contrasts with our relatively high estimate of K. This could result from a (plausibly adaptive: [47]) slowing (negative acceleration) of fetal growth prior to birth [48], which would lower perinatal estimates of TEE and K. To represent such a slowing, and a lower K (and H) at birth, the new composite model could be modified to include more than one *in utero* component growth process, and then fit to a dataset that includes fetal (and placental) size measures.

### 4.2. Inter-group comparisons

Figure 4 suggests that, just prior to birth, Matsigenka tend to have lower (boys) or indistinguishable (girls) average rates of catabolism (K) and anabolism (H) compared to U.S. children. However, by approximately two to five years after birth, Matsigenka metabolic rates are estimated to be higher than those of U.S. children, a pattern that persists into adulthood. Perinatal comparisons must be interpreted with caution, as *in utero* and infant component growth functions are fit to very few measurements of Matsigenka children younger than two years (electronic supplementary material, appendix Figure A.8). This means that Matsigenka estimates in these early life stages are more strongly influenced by the priors, which, in this case, are derived from U.S. children (electronic supplementary material, Appendices D.2 and D.3). Thus, Matsigenka-U.S. differences in early life may be even greater than estimated here, in contrast to studies in other populations that have found little cross-cultural variation in weight-adjusted infant energy expenditure [49]. With this caveat in mind, the postnatal increase in Matsigenka metabolic rates compared to U.S. children could result, in part, from Matsigenka infants consuming more energy during the first years of life, perhaps relating to the timing and type of supplemental foods introduced during infancy, practices that were changing rapidly in the U.S. of the late 1920s [50–52]. This hypothesis requires future systematic study. At the same time, Matsigenka infants may also expend relatively more energy due to immune system activation in response to parasites or other pathogens in the tropical forest environment ([53–55], though see [56]), e.g. intestinal pathogens accompanying the introduction of solid food.

The higher mean Matsigenka rates of catabolism (K) relative to U.S. children are estimated to continue through ontogeny (figure 4). As with infants, this could be due to Matsigenka immune system activation in response to tropical pathogens and parasites, which is associated with shorter stature in other Amazonian Indigenous populations [53,57]. Mean rates of anabolism (H) are also estimated to be higher for Matsigenka than for U.S. children. This suggests that Matsigenka synthesize more mass per unit intestinal surface, which, for a given intestine size, can be achieved through consumption of more energy-dense foods. This is unexpected, given that Matsigenka children’s foods are produced through their parents’ daily fishing, hunting, gathering and horticultural activities, that, on average, likely have more fiber and lower energy density than foods available to upper-class U.S. children in the 1930s, a time when commercially processed foods were gaining in popularity [50]. One potential explanation is that Matsigenka have greater intestinal surface area for their body size than do patients in Euro-American clinics from whom our surface area calculations are derived (electronic supplementary material, appendix D.1.2). Indeed, Weaver *et al*. [58] reports considerable variation in human small intestine length for a given height, which suggests that intestinal surface area may adjust during ontogeny as a plastic response to disease and dietary changes. Underestimating Matsigenka intestinal surface area s would lead to an overestimate of the anabolic rate H (equation (2.1). This hypothesis requires future study.

Figure 4 suggests that, starting around age 10, Matsigenka children have lower values of the allometric parameter q than do U.S. children. Because $r=h^{q}$ (equation (2.4)), this is interpreted to mean that, for a given height, Matsigenka have a narrower skeleton, defining a smaller abdominal cavity, housing an intestine with smaller surface area (electronic supplementary material, appendix D.1.1). This result coincides with our impression that Matsigenka tend to have ectomorphic bodies. However, it is unexpected given the presumably lower energy density (e.g. higher fiber content) of typical Matsigenka foods, and the negative relationship between gut surface area and dietary energy density (fauna > fruit > foliage) observed in other mammals [59]. Underestimation of the Matsigenka q parameter could be produced through a disproportionate underestimation of Matsigenka weight (electronic supplementary material, appendix G.3), or, as above for H, an underestimation of Matsigenka intestinal surface area for a given body size. A future challenge will be to determine (noninvasively) whether populations like the Matsigenka tend to surpass urban populations in intestinal surface area per abdominal volume.

### 4.3. Healthcare interventions

Figure 2 suggests that observed differences in stature between Matsigenka and U.S. children are primarily the result of component growth processes that make their most substantial contributions to overall height *in utero*, during infancy and during early childhood (i.e. the first three component growth processes). This suggests that, if such differences signal growth-related health challenges, interventions should be targeted toward children in these earlier developmental stages. This highlights an advantage of process-partitioned composite growth models, like that developed here (as well as the ICP [60] and QEPS models [18,61]): they allow exploration of the ages at which population-level differences in growth contribute to observed size differences. This is more difficult with other growth models (e.g. JPA-1: [15] and SITAR: [19]), that represent growth over all of ontogeny as a single process [62].

A unique advantage of the causal theoretical model developed here compared to previous models is the ability to simulate interventions, which can inspire hypotheses about underlying causes of growth variation, suggest which strategies may be most effective in alleviating health challenges, and guide expectations about what results of a successful healthcare strategy will look like. For example, figure 5 suggests that the shorter mean stature of Matsigenka children relative to U.S. children is partly due to metabolic differences between these populations. These may be responsive to environmental manipulation. For instance, Matsigenka are predicted to grow taller after an intervention that (i) increases anabolic rates (H), e.g. through the provision of supplemental foods dense in energy and substrates for corporal construction; and (ii) decreases catabolic rates (K), e.g. through disease treatment and prevention to decrease immune system activation. Importantly, however, this analysis suggests that allometry (q) also contributes to height differences between Matsigenka and U.S. children, as simulated elimination of average metabolic differences (e.g. through the healthcare intervention described above) is not predicted to equalize their average heights (figure 5). Compared to metabolism, allometry (body shape) may be under stronger genetic control, and thus more responsive to drift and natural selection [7] at the population level on generational time scales. For instance, there is genetic evidence that short stature may provide a selective advantage in tropical forest environments [6,63,64], though the nature of any such height adaptation, or linkage to another adaptive trait, is still poorly understood [65]. If allometric differences of genetic origin contribute substantially to population differences in average height, it would lend support to recent calls for caution [12,66] when diagnosing stunting, a form of malnutrition defined as low height for age, by comparing children’s growth against a single universal human standard, as is currently the norm in international contexts [14,67].

Interestingly, interventions on metabolic rates can affect overall body shape, even if the allometric q parameters of each component growth process are unchanged (e.g., under complete genetic control). Electronic supplementary material, appendix figure A.19 shows that a simulated intervention to equalize mean Matsigenka and U.S. metabolic rates results in nearly identical mean weight trajectories, even while height trajectories remain distinct (figure 5). This suggests that the intervention would cause Matsigenka to become heavier for a given height. Electronic supplementary material, appendix figure A.20 shows that the simulated intervention has indeed increased overall Matsigenka q, and by extension intestinal surface area and body circumference for a given height, above that of U.S. children. This effect is due to the fact that metabolic rates affect the shapes of the five component growth functions, and thus their weighted contributions to the overall value of q at a given age (electronic supplementary material, appendix E). Metabolism-induced changes in the shape of component growth functions result in a similar effect on the timing of growth: the simulated metabolic intervention decreases Matsigenka age at maximum pubertal growth velocity (compare electronic supplementary material, appendix figures A.10 and A.22), despite the fact that initiation parameters i for each component growth process are unchanged. These effects parallel Tanner *et al*. [9]’s analysis of a secular trend whereby growth patterns of cohorts of Japanese children changed over the course of 20 years, during which living (and presumably nutritional and metabolic) conditions improved: children’s legs (but not trunk) grew longer, and they reached maximum pubertal growth velocity earlier. In the context of our new model, we would hypothesize that improving living conditions in Japan increased anabolism (H) and decreased catabolism (K) in a component growth process that contributed more to leg length than to expansion of the abdominal cavity (and intestinal surface area), and thus had a low value of the allometric q parameter. Changing the shape of this component process (i.e. increasing its contribution to overall growth), relative to that of the other processes, resulted in a shallower overall q trajectory and an earlier timing of maximum pubertal growth.

Like nearly all phenotypic traits, both body proportions and metabolic rates are the product of interactions between genes and the environment. Although we assume that genetic contributions to body proportions (allometry) tend to be larger than environmental contributions, the above discussion suggests that this may not always be the case (see also [7]). Similarly, although immune system activation resulting from parasite and pathogen exposure is associated with higher metabolic rates [54], it may also be the case that genetic differences contribute substantially to inter-individual variation in metabolism. Future work to quantify the relative contributions of genes and the environment to allometry and metabolism could use the composite model to analyze growth patterns in a population requesting a specific healthcare intervention. Changes in individuals’ allometric and metabolic parameters in the years before and after the intervention could be attributable solely to the environmental manipulation, especially if the timescale is too short for drift and natural selection to change gene frequencies in the population. Such a study could test the validity of our assumption that allometric q parameters are under stronger genetic than environmental influence relative to metabolic parameters H and K.

### 4.4. Limitations and future directions

In developing this theory of human growth, we make a number of simplifying assumptions to facilitate derivation and fitting of the mathematical model. These assumptions, detailed in electronic supplementary material, appendices A–D, place important limits on interpretation of model estimates, and, at the same time, serve as a guide for future work. For instance, in the absence of cross-cultural data, we assume a constant and universal relationship between skin surface area and intestinal surface area (electronic supplementary material, appendix D.1.2), which, as explained above, may distort estimates of H and q for populations like the Matsigenka.

We represent components of the body as cylinders (electronic supplementary material, appendix figure A.1), the radii and heights of which are constrained to grow according to a simple power function ($r=h^{q}$, equation (2.4)). While better than alternative simple functions (electronic supplementary material, appendix figures A.2 and A.3), this assumption precludes any change in radius (and by extension, weight) resulting from soft tissue (e.g. fat and muscle) development that does not correspond to a height increase. When fitting the model simultaneously to both height and weight measurements, we compensate for this assumption by defining unequal constraints on variance due to measurement error between the two data types (electronic supplementary material, appendix D.1.3). Electronic supplementary material, appendices F.1 and F.2 present alternative, less satisfactory approaches. A mechanistic theory that explicitly incorporates soft tissue growth, and accounts for changes in body composition during ontogeny, would constitute an improvement over the present model.

In computing overall mean parameter values by age (figure 4), we assume that metabolic rates decrease as a component growth process approaches its asymptote, primarily due to replacement of metabolically active red marrow cells by less active marrow fat cells as the skeleton ages. In the absence of data, the proportion by which metabolic rates decrease at the asymptote is arbitrarily set to 0.75 (see electronic supplementary material, appendix D.4 for modification of this assumption). Future work is needed to determine if, and by how much, mean rates of anabolism and catabolism change with the replacement of such cell types during ontogeny.

In this study, Matsigenka children are represented by a temporally-sparse dataset (electronic supplementary material, appendix figure A.8) typical of those collected in remote non-urban societies often neglected in clinical growth studies. While the Bayesian analytical techniques employed here facilitate estimation of growth trajectories from sparse datasets, more longitudinal data usually result in more accurate estimates. Thus, it will be of interest to know if and how model estimates of Matsigenka growth parameters change as more data are collected in coming years. Given the sparse dataset, and the many assumptions of this new growth model that have yet to be systematically explored, the above comparison of U.S. and Matsigenka children’s growth should not be viewed as a definitive analysis upon which to base healthcare decisions. Rather, it illustrates the promise and potential of this new approach to serve as a framework to: (i) relate height, weight, and intestinal growth to body allometry and rates of anabolism and catabolism; (ii) determine the relative contributions of metabolism (likely strongly affected by diet and disease) and allometry (likely more strongly affected by genetic factors) to observed size differences between individuals and populations; (iii) simulate the effect of healthcare interventions on children’s growth; and (iv) complement the strengths of existing growth models, such as SITAR [19], QEPS [18] and JPA-1 [15], by providing a theoretical foundation for the design of desired healthcare support strategies better tailored to the needs of particular communities, and, importantly, to guide exploration of the causes of growth variation in our species.

## Data Availability

All data, analysis scripts in R and Stan, and instructions to perform all analyses in the main text and the appendices are available on GitHub [68] and also on the Open Science Framework [69]. Supplementary material containing all appendices is available online [70].

## References

[1] von Bertalanffy L. 1957 Quantitative laws in metabolism and growth. Q. Rev. Biol. **32**, 217–231. (10.1086/401873) 13485376

[2] Bogin B. 1999 Evolutionary perspective on human growth. Annu. Rev. Anthropol. **28**, 109–153. (10.1146/annurev.anthro.28.1.109) 12295621

[3] Hamada Y, Udono T. 2002 Longitudinal analysis of length growth in the chimpanzee (Pan troglodytes). Am. J. Phys. Anthropol. **118**, 268–284. (10.1002/ajpa.10078) 12115283

[4] Eveleth PB, Tanner JM. 1990 Worldwide Variation in Human Growth, 2nd edn. Cambridge, UK: Cambridge University Press.

[5] Lello L, Avery SG, Tellier L, Vazquez AI, de Los Campos G, Hsu SDH. 2018 Accurate genomic prediction of human height. Genetics **210**, 477–497. (10.1534/genetics.118.301267) 30150289 PMC6216598

[6] Zoccolillo M, Moia C, Comincini S, Cittaro D, Lazarevic D, Pisani KA, Wit JM, Bozzola M. 2020 Identification of novel genetic variants associated with short stature in a Baka Pygmies population. Hum. Genet. **139**, 1471–1483. (10.1007/s00439-020-02191-x) 32583022 PMC7519921

[7] Katzmarzyk PT, Leonard WR. 1998 Climatic influences on human body size and proportions: ecological adaptations and secular trends. Am. J. Phys. Anthropol. **106**, 483–503. (10.1002/(sici)1096-8644(199808)106:43.3.co;2-k) 9712477

[8] Stewart CP, Iannotti L, Dewey KG, Michaelsen KF, Onyango AW. 2013 Contextualising complementary feeding in a broader framework for stunting prevention. Matern. Child Nutr. **9**, 27–45. (10.1111/mcn.12088) 24074316 PMC6860787

[9] Tanner JM, Hayashi T, Preece MA, Cameron N. 1982 Increase in length of leg relative to trunk in Japanese children and adults from 1957 to 1977: comparison with British and with Japanese Americans. Ann. Hum. Biol. **9**, 411–423. (10.1080/03014468200005951) 7137939

[10] Bogin B. 2022 Fear, violence, inequality, and stunting in Guatemala. Am. J. Hum. Biol. **34**, e23627. (10.1002/ajhb.23627) 34125987

[11] Li Z, Kim R, Vollmer S, Subramanian SV. 2020 Factors associated with child stunting, wasting, and underweight in 35 low- and middle-income countries. JAMA Netw. Open **3**, e203386. (10.1001/jamanetworkopen.2020.3386) 32320037 PMC7177203

[12] Hruschka DJ. 2021 One size does not fit all. How universal standards for normal height can hide deprivation and create false paradoxes. Am. J. Hum. Biol. **33**, e23552. (10.1002/ajhb.23552) 33314421

[13] World Health Organization, United Nations Children’s Fund. 2009 WHO child growth standards and the identification of severe acute malnutrition in infants and children: a joint statement by the World Health Organization and the United Nations Children’s Fund. Geneva, Switzerland: World Health Organization and UNICEF. See https://www.who.int/publications/i/item/9789241598163. 24809116

[14] de Onis M, Branca F. 2016 Childhood stunting: a global perspective. Matern. Child Nutr. **12**, 12–26. (10.1111/mcn.12231) 27187907 PMC5084763

[15] Jolicoeur P, Pontier J, Abidi H. 1992 Asymptotic models for the longitudinal growth of human stature. Am. J. Hum. Biol. **4**, 461–468. (10.1002/ajhb.1310040405) 28524389

[16] Hauspie RC, Molinari L. 2004 Parametric models for postnatal growth. In Methods in human growth research (eds RC Hauspie, N Cameron, L Molinari), pp. 205–233. Cambridge, UK: Cambridge University Press. (10.1017/CBO9780511542411.009)

[17] Gasser T, Gervini D, Molinari L. 2004 Kernel estimation, shape-invariant modelling and structural analysis. In Methods in human growth research (eds RC Hauspie, N Cameron, L Molinari), pp. 179–204. Cambridge, UK: Cambridge University Press. (10.1017/CBO9780511542411.008)

[18] Nierop AFM, Niklasson A, Holmgren A, Gelander L, Rosberg S, Albertsson-Wikland K. 2016 Modelling individual longitudinal human growth from fetal to adult life - QEPS I. J. Theor. Biol. **406**, 143–165. (10.1016/j.jtbi.2016.06.007) 27297288

[19] Cole TJ, Donaldson MDC, Ben-Shlomo Y. 2010 SITAR—a useful instrument for growth curve analysis. Int. J. Epidemiol. **39**, 1558–1566. (10.1093/ije/dyq115) 20647267 PMC2992626

[20] Lampl M. 2012 Perspectives on modelling human growth: mathematical models and growth biology. Ann. Hum. Biol. **39**, 342–351. (10.3109/03014460.2012.704072) 22834928

[21] Suki B, Frey U. 2017 A time-varying biased random walk approach to human growth. Sci. Rep. **7**, 7805. (10.1038/s41598-017-07725-4) 28798412 PMC5552693

[22] Pütter A. 1920 Studien über physiologische Ähnlichkeit VI. Wachstumsähnlichkeiten. Pflüger’s Arch. für die Gesamte Physiol. des Menschen und der Tiere **180**, 298–340. (10.1007/BF01755094)

[23] von Bertalanffy L. 1938 A quantitative theory of organic growth (Inquiries on growth laws. II). Hum. Biol. **10**, 181–213.

[24] Kooijman S, S.A.L.M. 2010 Dynamic Energy Budget Theory for Metabolic Organisation, 3rd edn. Cambridge, UK: Cambridge University Press. 10.1111/j.1469-185X.2006.00006.x 17313526

[25] Kooijman S, S.A.L.M. 2001 Quantitative aspects of metabolic organization: a discussion of concepts. Phil. Trans. R. Soc. Lond. B Biol. Sci. **356**, 331–349. (10.1098/rstb.2000.0771) 11316483 PMC1088431

[26] Gillooly JF, Brown JH, West GB, Savage VM, Charnov EL. 2001 Effects of size and temperature on metabolic rate. Science **293**, 2248–2251. (10.1126/science.1061967) 11567137

[27] West GB, Brown JH, Enquist BJ. 1997 A general model for the origin of allometric scaling laws in biology. Science **276**, 122–126. (10.1126/science.276.5309.122) 9082983

[28] West GB, Brown JH, Enquist BJ. 2001 A general model for ontogenetic growth. Nature **413**, 628–631. (10.1038/35098076) 11675785

[29] Tuddenham RD, Snyder MM. 1954 Physical growth of California boys and girls from birth to eighteen years. In University of California Publications in Child Development (eds HE Jones, C Landreth, JW MacFarlane), pp. 183–364, vol. 1. Berkeley, CA, USA: University of California Press. 13217130

[30] McElreath R. 2020 Statistical Rethinking: A Bayesian Course with Examples in R and Stan, 2nd edn. Boca Raton, FL, USA: CRC Press.(Texts in Statistical Science).

[31] Vincenzi S, Jesensek D, Crivelli AJ. 2020 Biological and statistical interpretation of size-at-age, mixed-effects models of growth. R. Soc. Open Sci. **7**, 192146. (10.1098/rsos.192146) 32431890 PMC7211857

[32] Johnson W. 2015 Analytical strategies in human growth research. Am. J. Hum. Biol. **27**, 69–83. (10.1002/ajhb.22589) 25070272 PMC4309180

[33] Butler G, McKie M, Ratcliffe S. 1990 The cyclical nature of prepubertal growth. Ann. Hum. Biol. **17**, 177–198. (10.1080/03014469000000952) 2337324

[34] Togo M, Togo T. 1982 Time-series analysis of stature and body weight in five siblings. Ann. Hum. Biol. **9**, 425–440. (10.1080/03014468200005961) 7137940

[35] Lampl M, Veldhuis JD, Johnson ML. 1992 Saltation and stasis: a model of human growth. Science **258**, 801–803. (10.1126/science.1439787) 1439787

[36] Lampl M, Johnson M. 1993 A case study of daily growth during adolescence: a single spurt or changes in the dynamics of saltatory growth? Ann. Hum. Biol. **20**, 595–603. (10.1080/03014469300003002) 8257085

[37] Knuth DE. 1992 Two notes on notation. Am. Math. Mon. **99**, 403–422. (10.1080/00029890.1992.11995869)

[38] Bunce JA, McElreath R. 2017 Interethnic interaction, strategic bargaining power, and the dynamics of cultural norms: a field study in an Amazonian population. Hum. Nat. **28**, 434–456. (10.1007/s12110-017-9297-8) 28822079 PMC5662675

[39] Revilla Minaya C. 2019 Environmental factishes, variation, and emergent ontologies among the Matsigenka of the Peruvian Amazon. Ph.D. thesis. Vanderbilt University. See https://www.proquest.com/docview/2243786665?fromopenview=true&fromunauthdoc=true&pq-origsite=gscholar&sourcetype=Dissertations%20&%20Theses.

[40] Shepard GH, Rummenhoeller K, Ohl-Schacherer J, Yu DW. 2010 Trouble in paradise: Indigenous populations, anthropological policies, and biodiversity conservation in Manu National Park, Peru. J. Sustain. For. **29**, 252–301. (10.1080/10549810903548153)

[41] Leary C, Leese HJ, Sturmey RG. 2014 Human embryos from overweight and obese women display phenotypic and metabolic abnormalities. Hum. Reprod. **30**, 122–132. (10.1093/humrep/deu276) 25391239

[42] Hatton IA, Galbraith ED, Merleau NSC, Miettinen TP, Smith BM, Shander JA. 2023 The human cell count and size distribution. Proc. Natl Acad. Sci. USA **120**, e2303077120. (10.1073/pnas.2303077120) 37722043 PMC10523466

[43] R Core Team. 2022 R: a language and environment for statistical computing. Vienna, Austria: R Foundation for Statistical Computing. See https://www.R-project.org/.

[44] Stan Development Team. 2022 Stan modeling language: user’s guide and reference manual, version 2.30. See https://mc-stan.org/docs/2_30/stan-users-guide-2_30.pdf.

[45] Gabry J, Cesnovar R. 2021 cmdstanr: R interface to ‘CmdStan’. See https://mc-stan.org/cmdstanr.

[46] Pontzer H *et al*. 2021 Daily energy expenditure through the human life course. Science **373**, 808–812. (10.1126/science.abe5017) 34385400 PMC8370708

[47] Vasak B, Koenen SV, Koster MPH, Hukkelhoven CWPM, Franx A, Hanson MA, Visser GHA. 2015 Human fetal growth is constrained below optimal for perinatal survival. Ultrasound Obstet. Gynecol. **45**, 162–167. (10.1002/uog.14644) 25092251

[48] Kiserud T, Benachi A, Hecher K, Perez RG, Carvalho J, Piaggio G, Platt LD. 2018 The World Health Organization fetal growth charts: concept, findings, interpretation, and application. Am. J. Obstet. Gynecol. **218**, S619–S629. (10.1016/j.ajog.2017.12.010) 29422204

[49] Prentice AM, Paul AA. 2000 Fat and energy needs of children in developing countries. Am. J. Clin. Nutr. **72**, 1253S–1265S. (10.1093/ajcn/72.5.1253s) 11063467

[50] Bentley A. 2014 Inventing Baby Food: Taste, Health, and the Industrialization of the American Diet. Oakland, CA, USA: California Studies in Food and Culture, University of California Press.

[51] Castilho SD, Filho AdA. 2010 The history of infant nutrition. J. Pediatr. **86**, 179–188. (10.2223/JPED.1984) 20520922

[52] Stevens EE, Patrick TE, Pickler R. 2009 A history of infant feeding. J. Perinat. Educ. **18**, 32–39. (10.1624/105812409X426314) PMC2684040 20190854

[53] Urlacher SS, Ellison PT, Sugiyama LS, Pontzer H, Eick G, Liebert MA, Cepon-Robins TJ, Gildner TE, Snodgrass JJ. 2018 Tradeoffs between immune function and childhood growth among Amazonian forager-horticulturalists. Proc. Natl Acad. Sci. USA **115**, E3914–E3921. (10.1073/pnas.1717522115) 29632170 PMC5924892

[54] Gurven MD, Trumble BC, Stieglitz J, Yetish G, Cummings D, Blackwell AD, Beheim B, Kaplan HS, Pontzer H. 2016 High resting metabolic rate among Amazonian forager-horticulturalists experiencing high pathogen burden. Am. J. Phys. Anthropol. **161**, 414–425. (10.1002/ajpa.23040) 27375044 PMC5075257

[55] Garcia AR, Blackwell AD, Trumble BC, Stieglitz J, Kaplan H, Gurven MD. 2020 Evidence for height and immune function trade-offs among preadolescents in a high pathogen population. Evol. Med. Public Health **2020**, 86–99. (10.1093/emph/eoaa017) 32983534 PMC7502263

[56] Urlacher SS, Snodgrass JJ, Dugas LR, Madimenos FC, Sugiyama LS, Liebert MA, Joyce CJ, Terán E, Pontzer H. 2021 Childhood daily energy expenditure does not decrease with market integration and is not related to adiposity in Amazonia. J. Nutr. **151**, 695–704. (10.1093/jn/nxaa361) 33454748

[57] McDade TW, Reyes-García V, Tanner S, Huanca T, Leonard WR. 2008 Maintenance versus growth: investigating the costs of immune activation among children in lowland Bolivia. Am. J. Phys. Anthropol. **136**, 478–484. (10.1002/ajpa.20831) 18383156

[58] Weaver LT, Austin S, Cole TJ. 1991 Small intestinal length: a factor essential for gut adaptation. Gut **32**, 1321–1323. (10.1136/gut.32.11.1321) 1752463 PMC1379160

[59] Chivers DJ, Hladik CM. 1980 Morphology of the gastrointestinal tract in primates: comparisons with other mammals in relation to diet. J. Morphol. **166**, 337–386. (10.1002/jmor.1051660306) 7441763

[60] Karlberg J. 1989 A biologically-oriented mathematical model (ICP) for human growth. Acta Paediatr. **78**, 70–94. (10.1111/j.1651-2227.1989.tb11199.x) 2801108

[61] Holmgren A, Niklasson A, Nierop AFM, Butler G, Albertsson-Wikland K. 2022 Growth pattern evaluation of the Edinburgh and Gothenburg cohorts by QEPS height model. Pediatr. Res. **92**, 592–601. (10.1038/s41390-021-01790-2) 34732814

[62] Mansukoski L, Johnson W, Brooke-Wavell K, Galvez-Sobral JA, Furlan L, Cole TJ, Bogin B. 2019 Life course associations of height, weight, fatness, grip strength, and all-cause mortality for high socioeconomic status Guatemalans. Am. J. Hum. Biol. **31**, e23253. (10.1002/ajhb.23253) 31090124 PMC6767560

[63] Perry GH *et al*. 2014 Adaptive, convergent origins of the pygmy phenotype in African rainforest hunter-gatherers. Proc. Natl Acad. Sci. USA **111**, E3596–603. (10.1073/pnas.1402875111) 25136101 PMC4156716

[64] Perry GH, Verdu P. 2017 Genomic perspectives on the history and evolutionary ecology of tropical rainforest occupation by humans. Quat. Int. **448**, 150–157. (10.1016/j.quaint.2016.04.038)

[65] Perry GH, Dominy NJ. 2009 Evolution of the human pygmy phenotype. Trends Ecol. Evol. **24**, 218–225. (10.1016/j.tree.2008.11.008) 19246118

[66] Hackman JV, Hruschka DJ. 2020 Disentangling basal and accrued height-for-age for cross-population comparisons. Am. J. Phys. Anthropol. **171**, 481–495. (10.1002/ajpa.23990) 31886899

[67] de Onis M, WHO Multicentre Growth Reference Study Group. 2006 Assessment of differences in linear growth among populations in the WHO Multicentre Growth Reference Study. Acta Paediatr. **95**, 56–65. (10.1111/j.1651-2227.2006.tb02376.x) 16817679

[68] jabunce. 2025 bunce-fernandez-revilla-2022-growth-model. https://github.com/jabunce/bunce-fernandez-revilla-2022-growth-model

[69] Bunce JA. 2025 A causal model of human growth and its estimation using temporally-sparse data. (10.17605/OSF.IO/KG5WN) PMC12329442 40777922

[70] Bunce J, Fernández C, Revilla-Minaya C. 2025 Supplementary material from: A causal model of human growth and its estimation using temporally-sparse data. Figshare. (10.6084/m9.figshare.c.7881967) PMC12329442 40777922

